# Supplementary material for: Effects of esketamine on postoperative pain, anxiety, depression, sleep, and inflammation in pregnancies undergoing cesarean section: A randomized controlled trial
Source: PLoS One. 2026 Jan 23;21(1):e0328585. doi: 10.1371/journal.pone.0328585 (PMC12829813; doi:10.1371/journal.pone.0328585)
Supplement: S2 File — (DOCX) [file pone.0328585.s003.docx]

## Randomized Controlled Study on S-Ketamine Alleviating Post-Cesarean Delivery Pain Hypersensitivity

### I. Research Background and Justification

**Research significance, current status, and trends in domestic and international research, analyzed in the context of scientific development trends.** (Attached main reference directory)

Cesarean delivery (CD) is one of the primary methods of childbirth for pregnant women. In recent years, the rate of cesarean deliveries in China has gradually increased. From 2008 to 2014, the cesarean delivery rate rose from 28.8% to 34.9%. The China Maternal and Child Health Development Report (2019) indicated that the cesarean delivery rate in China further increased to 36.7% in 2018. Postoperative pain is a topic of international concern and the primary concern for women undergoing CD.^[1]^ Reports indicate that 39%-78% of women experience acute moderate-to-severe pain within 24 hours after CD.^[2, 3]^ Lavand'homme, P. noted that 6.1%-11.5% of these cases develop into chronic pain syndromes.^[4]^ Poor pain control not only adversely affects early postoperative mobility, breastfeeding, and mother-infant bonding^[5]^ but is also associated with various adverse outcomes such as postpartum depression, myocardial infarction, pulmonary infections, gastrointestinal motility reduction, nausea and vomiting, impaired immune function, and delayed wound healing.^[6-8]^ Pain hypersensitivity refers to a reduced pain threshold, an expanded range of pain perception, and even contact pain in areas distant from the surgical site, which is a significant cause of acute and chronic postoperative pain. **Therefore, effectively reducing postoperative pain hypersensitivity after CD and subsequently lowering the incidence of acute and chronic pain has become an urgent clinical issue.**

Currently, there is no effective clinical method to address postoperative pain hypersensitivity after CD. The commonly used postoperative analgesia is Patient-Controlled Intravenous Analgesia (PCIA), which involves adding opioids to an analgesic pump set at a background dose for continuous hourly infusion. If the patient experiences unbearable pain, they can press the pump button to receive an additional dose of analgesic. However, opioids used in PCIA not only cause side effects such as nausea, vomiting, urinary retention, and respiratory depression but can also exacerbate pain hypersensitivity, reducing their analgesic efficacy. High doses may lead to tolerance and addiction.^[9]^

In preliminary experiments, mechanical pain sensitivity measurements before and after cesarean delivery revealed a significant increase in mechanical pain sensitivity postpartum, indicating pronounced pain hypersensitivity. Trauma-induced inflammatory responses, opioid use for postoperative analgesia, sleep disturbances, and anxiety/depression can all contribute to pain hypersensitivity. Trauma surrounding tissues generates necrotic substances, leading to the production of various pro-inflammatory factors that directly or indirectly act on nociceptive sensory neurons, lowering the excitation threshold of peripheral neurons and causing peripheral sensitization. Persistent peripheral sensitization activates Aδ and C fibers, transmitting nociceptive signals to the spinal dorsal horn and promoting the expression of pro-inflammatory factors in the spinal cord, resulting in the release of large amounts of glutamate. Glutamate further activates N-methyl-D-aspartate (NMDA) receptors on postsynaptic membranes, altering spinal synaptic plasticity and transmitting pain signals to the cerebral cortex, leading to central sensitization.^[10]^ Central and peripheral sensitization together constitute pain hypersensitivity. Opioids can also activate NMDA receptors on spinal dorsal horn neurons, increasing neuronal excitability and enhancing pain sensitivity in surrounding tissues and the central nervous system, forming pain hypersensitivity.^[9]^ Additionally, multiple studies have confirmed that perioperative sleep disturbances and anxiety/depression can induce pain hypersensitivity.^[11-16]^ **Thus, NMDA receptor activation is a critical node in the formation of pain hypersensitivity.**^[17]^ **Antagonizing NMDA receptors to inhibit their activation may be an effective approach to alleviating postoperative pain after cesarean delivery.**

S-Ketamine is a newly developed and clinically applied adjuvant intravenous anesthetic. As an optical isomer of ketamine, it non-competitively antagonizes NMDA receptors, inhibiting the release of excitatory neurotransmitters (including acetylcholine and L-glutamate) in the brain to produce anesthetic effects.^[18]^ Tu, W. et al. confirmed that S-Ketamine also exerts strong anti-inflammatory effects by inhibiting the release of inflammatory cells stimulated by oxygen free radicals and reducing the secretion of interleukins and tumor necrosis factor by leukocytes.^[19]^ Multiple studies have demonstrated that S-Ketamine can improve postoperative sleep and reduce the incidence of anxiety and depression.^[20-25]^ **Therefore, S-Ketamine may have potential effects in inhibiting the formation of pain hypersensitivity.**

Previous studies have established that ketamine can inhibit pain hypersensitivity. S-Ketamine, as an optical isomer of ketamine, shares similar pharmacological properties.^[26-29]^ Several studies have also shown that ketamine used as an adjuvant anesthetic in cesarean delivery produces good analgesic effects.^[30-33]^ Yan-Ling Ren et al. reported that S-Ketamine could inhibit pain hypersensitivity after thyroidectomy.^[34]^ Yongjian Lu et al. found that S-Ketamine effectively reduced postoperative pain hypersensitivity and delirium in elderly patients undergoing thoracic surgery.^[35]^ However, there are currently no reports on the effects of S-Ketamine on pain hypersensitivity after cesarean delivery. Therefore, this study aims to explore the impact of S-Ketamine administered during the perioperative period of cesarean delivery on postoperative pain hypersensitivity. The findings may further optimize anesthesia protocols for cesarean delivery, alleviating postoperative pain and promoting rapid recovery.

**References:**

### 1. Sutton, C.D. and B. Carvalho, Optimal Pain Management After Cesarean Delivery. Anesthesiol Clin, 2017. 35(1): p. 107-124.

### 2. Bjornstad, J. and J. Raeder, Post-operative pain after caesarean section. Tidsskr Nor Laegeforen, 2020. 140(7).

### 3. Kintu, A., et al., Postoperative pain after cesarean section: assessment and management in a tertiary hospital in a low-income country. BMC Health Serv Res, 2019. 19(1): p. 68.

### 4. Lavand'homme, P., Postpartum chronic pain. Minerva Anestesiol, 2019. 85(3): p. 320-324.

### 5. Xu, M., et al., Quadratus lumborum block for postoperative analgesia after cesarean delivery: a systematic review and meta-analysis. Int J Obstet Anesth, 2020. 42: p. 87-98.

### 6. Gan, T.J., Poorly controlled postoperative pain: prevalence, consequences, and prevention. J Pain Res, 2017. 10: p. 2287-2298.

### 7. Yang, M.M.H., et al., Preoperative predictors of poor acute postoperative pain control: a systematic review and meta-analysis. BMJ Open, 2019. 9(4): p. e025091.

### 8. Yimer, H. and H. Woldie, Incidence and Associated Factors of Chronic Pain After Caesarean Section: A Systematic Review. J Obstet Gynaecol Can, 2019. 41(6): p. 840-854.

### 9. Colvin, L.A., F. Bull, and T.G. Hales, Perioperative opioid analgesia—when is enough too much? A review of opioid-induced tolerance and hyperalgesia. The Lancet, 2019. 393(10180): p. 1558-1568.

### 10. Meng, Y., et al., NLRP3-mediated Neuroinflammation Exacerbates Incisional Hyperalgesia and Prolongs Recovery After Surgery in Chronic Stressed Rats. Pain Physician, 2021. 24(7): p. E1099-E1108.

### 11. Huang, Y., et al., Pretreatment of the ROS Inhibitor Phenyl-N-tert-butylnitrone Alleviates Sleep Deprivation-Induced Hyperalgesia by Suppressing Microglia Activation and NLRP3 Inflammasome Activity in the Spinal Dorsal Cord. Neurochemical Research, 2022. 48(1): p. 305-314.

### 12. Zhao, J., et al., Depression comorbid with hyperalgesia: Different roles of neuroinflammation induced by chronic stress and hypercortisolism. Journal of Affective Disorders, 2019. 256: p. 117-124.

### 13. Piardi, L.N., et al., Social stress as a trigger for depressive-like behavior and persistent hyperalgesia in mice: study of the comorbidity between depression and chronic pain. Journal of Affective Disorders, 2020. 274: p. 759-767.

### 14. Fülöp, B., et al., IL-1 Mediates Chronic Stress-Induced Hyperalgesia Accompanied by Microglia and Astroglia Morphological Changes in Pain-Related Brain Regions in Mice. International Journal of Molecular Sciences, 2023. 24(6).

### 15. Yu, Z., et al., Acute sleep deprivation aggravates nitroglycerin-evoked hyperalgesia in mice. Molecular Pain, 2023. 19.

### 16. Guo, M., et al., Preoperative Acute Sleep Deprivation Causes Postoperative Pain Hypersensitivity and Abnormal Cerebral Function. Neurosci Bull, 2022. 38(12): p. 1491-1507.

### 17. Thompson, T., et al., NMDA receptor antagonists and pain relief: A meta-analysis of experimental trials. Neurology, 2019. 92(14): p. e1652-e1662.

### 18. Schwenk, E.S., et al., Ketamine in the Past, Present, and Future: Mechanisms, Metabolites, and Toxicity. Curr Pain Headache Rep, 2021. 25(9): p. 57.

### 19. Tu, W., et al., Influence of anesthetic induction of propofol combined with esketamine on perioperative stress and inflammatory responses and postoperative cognition of elderly surgical patients. Am J Transl Res, 2021. 13(3): p. 1701-1709.

### 20. Qiu, D., et al., Effect of Intraoperative Esketamine Infusion on Postoperative Sleep Disturbance After Gynecological Laparoscopy: A Randomized Clinical Trial. JAMA Netw Open, 2022. 5(12): p. e2244514.

### 21. Borentain, S., et al., Effect of Sleep Disturbance on Efficacy of Esketamine in Treatment-Resistant Depression: Findings from Randomized Controlled Trials. Neuropsychiatr Dis Treat, 2021. 17: p. 3459-3470.

### 22. Hu, N., et al., Exploring the role of esketamine in alleviating depressive symptoms in mice via the PGC-1alpha/irisin/ERK1/2 signaling pathway. Sci Rep, 2023. 13(1): p. 16611.

### 23. Kitay, B.M., et al., Cognitive behavioral therapy following esketamine for major depression and suicidal ideation for relapse prevention: The CBT-ENDURE randomized clinical trial study protocol. Psychiatry Res, 2023. 330: p. 115585.

### 24. Guo, Y., et al., Analgesic Effect of Esketamine Combined with Tramadol for Patient-Controlled Intravenous Analgesia After Cesarean Section: A Randomized Controlled Trial. J Pain Res, 2023. 16: p. 3519-3528.

### 25. Thornton, N.L.R., et al., Establishing an esketamine clinic in Australia: Practical recommendations and clinical guidance from an expert panel. Asia Pac Psychiatry, 2023. 15(4): p. e12550.

### 26. Hayhurst, C.J., E. Farrin, and C.G. Hughes, The effect of ketamine on delirium and opioid-induced hyperalgesia in the Intensive Care Unit. Anaesth Crit Care Pain Med, 2018. 37(6): p. 525-527.

### 27. Delgado, M.A., et al., Preclinical study in a postoperative pain model to investigate the action of ketamine, lidocaine, and ascorbic acid in reversing fentanyl-induced, non-glutamate-dependent hyperalgesia. Pain Rep, 2023. 8(2): p. e1062.

### 28. Zhou, X., et al., A single dose of ketamine relieves fentanyl-induced-hyperalgesia by reducing inflammation initiated by the TLR4/NF-kappaB pathway in rat spinal cord neurons. Drug Discov Ther, 2023. 17(4): p. 279-288.

### 29. Martinez, M.R., et al., Ketamine as an Analgesic Adjunct for Opioid-Induced Hyperalgesia in a Patient With a Sickle Cell Pain Episode. Ochsner J, 2022. 22(3): p. 281-284.

### 30. Wang, J., et al., Impact of Ketamine on Pain Management in Cesarean Section: A Systematic Review and Meta-Analysis. Pain Physician, 2020. 23(2): p. 135-148.

### 31. Samuel, H., S. Aweke, and J. Tuni, Effect of low-dose intravenous ketamine on postoperative pain following cesarean section under spinal anesthesia: A prospective cohort study, Ethiopia. Ann Med Surg (Lond), 2022. 77: p. 103570.

### 32. Haghighi, M., et al., The utero-tonic effects of low dose intravenous ketamine in cesarean section under spinal anesthesia; A randomized double-blind clinical trial. Caspian J Intern Med, 2023. 14(2): p. 218-225.

### 33. Resch, S.C., et al., Non-anaesthetist-administered ketamine for emergency caesarean section in Kenya: cost-effectiveness analysis. BMJ Open, 2022. 12(10): p. e051055.

### 34. Ren, Y.L., et al., Effects of Different Doses of Esketamine on Pain Sensitivity of Patients Undergoing Thyroidectomy: A Randomized Controlled Trial. Pain Ther, 2023. 12(3): p. 739-750.

### 35. Lu, Y., et al., The Application Value of Esketamine and Dexmedetomidine in Preventing Postoperative Delirium and Hyperalgesia in Elderly Patients with Thoracic Anesthesia. Altern Ther Health Med, 2023.

### II. Research Content and Objectives

**Describe the research content, objectives, and key scientific questions to be addressed.**

**(1) Research Content:**

1. Enroll women scheduled for elective cesarean delivery under combined spinal-epidural anesthesia and design a prospective randomized controlled parallel trial. Administer S-Ketamine perioperatively, using placebo and conventional PCIA as controls. Compare the effects of combined S-Ketamine on postoperative pain hypersensitivity by measuring changes in pressure pain thresholds and tolerance before and after surgery.
2. Collect blood samples from each group before and after surgery to measure serum C-reactive protein (CRP) levels. Compare differences in concentration changes to explore the impact of S-Ketamine on postoperative inflammatory responses. Combine assessments of sleep, anxiety, and depression to investigate the potential mechanisms by which S-Ketamine alleviates postoperative pain hypersensitivity. Confirm the efficacy of S-Ketamine in reducing postoperative pain through pain scores.

**(2) Research Objectives:**

1. Verify that perioperative application of S-Ketamine can effectively reduce postoperative pain hypersensitivity after cesarean delivery.
2. Compare the effects of perioperative S-Ketamine on inflammation, sleep, anxiety, and depression to explore its potential mechanisms in alleviating postoperative pain.
3. Confirm that S-Ketamine can effectively relieve postoperative pain after cesarean delivery.

**(3) Key Scientific Questions to Be Addressed:**

1. **Post-cesarean pain hypersensitivity:** Pain hypersensitivity is a major cause of postoperative pain. Poor pain control can severely affect maternal recovery and mother-infant bonding. Mechanistically, S-Ketamine has the potential to alleviate postoperative pain hypersensitivity. This study will validate whether perioperative S-Ketamine can effectively reduce postoperative pain hypersensitivity by measuring changes in pressure pain thresholds and tolerance before and after surgery.
2. **Mechanisms of S-Ketamine in alleviating pain hypersensitivity:** Basic research confirms that pain hypersensitivity is closely related to surgical-induced inflammatory responses. This study will simultaneously measure serum CRP levels before and after surgery to explore the potential mechanisms by which S-Ketamine improves postoperative analgesia. Postoperative pain scores and analgesic drug usage will also be evaluated.

### III. Research Plan

**Including research methods, technical routes, experimental approaches, key technologies, feasibility analysis, and the unique innovations of this project.**

**(1) Research Subjects:**

**Study Population:** Women scheduled for elective cesarean delivery under combined spinal-epidural anesthesia at our hospital.

**(2) Inclusion Criteria:**

1. Aged 20-45 years;
2. Scheduled for elective cesarean delivery under neuraxial anesthesia;
3. ASA physical status II-III;
4. Willing to participate and sign informed consent.

**(3) Exclusion Criteria:**

1. Contraindications for cesarean delivery (e.g., intrauterine fetal death, fetal malformation, maternal intolerance to surgery, fetal distress);
2. Contraindications for neuraxial anesthesia (e.g., coagulation disorders, central nervous system diseases);
3. Contraindications for S-Ketamine (e.g., hyperthyroidism, severe hypertension, preeclampsia, psychiatric disorders, or conditions requiring uterine relaxation such as umbilical cord prolapse or uterine rupture);
4. Alcohol abuse or long-term use of opioids, hormonal drugs, or anti-inflammatory analgesics;
5. Allergy to study drugs;
6. Participation in other clinical trials or use of other investigational drugs within three months;
7. Inability to cooperate or refusal to participate.

**(4) Elimination Criteria:**

1. No use of study drugs (e.g., withdrawal of consent before intervention);
2. No recorded measurements (e.g., canceled surgery).

Eliminated cases must be documented with reasons and excluded from efficacy analysis, but their CRF forms will be retained for reference.

**(5) Withdrawal (Dropout) Criteria:**

1. **Investigator-initiated withdrawal:** Participants may be withdrawn if they:
   - Cannot cooperate;
   - Experience worsening conditions requiring emergency measures;
   - Develop severe complications making continuation inappropriate.
2. **Participant-initiated withdrawal:** Participants may voluntarily withdraw.
3. **Drug discontinuation due to adverse events.**

For withdrawn participants, investigators must document reasons in the CRF and complete all possible assessments. Eliminated cases must be documented with reasons, and their CRF forms retained.

**(6) Experimental Methods:**

1. This study is designed as a prospective randomized controlled double-blind parallel trial, following CONSORT guidelines. Participants will be enrolled sequentially.
2. **Randomization:** Random numbers will be generated using SPSS software and sealed in sequentially numbered envelopes. An independent researcher will allocate drugs based on envelope numbers, which will remain sealed until study completion.
3. **Blinding:** Anesthesiologists managing intraoperative anesthesia and data recording, as well as trained researchers conducting perioperative assessments and analgesic pump data collection, will be blinded to group allocation. Statistical analysis will be performed by the Statistics Department of Fuling Hospital, Chongqing University. All participants will receive uniformly appearing drugs and PCIA pumps.
4. **Preoperative Data Collection:**
   - Demographic data (age, height, weight, gestational age);
   - Medical history (current/past illnesses, surgical/anesthesia history, ASA classification);
   - Mental status: Assessed using Generalized Anxiety Disorder-7 (GAD-7) and Edinburgh Postnatal Depression Scale (EPDS);
   - Sleep quality: Assessed using Pittsburgh Sleep Quality Index (PSQI);
   - Serum CRP levels;
   - Pressure pain threshold and tolerance.
5. **Anesthesia Procedure:**
   - Combined spinal-epidural anesthesia at L2-L3 or L3-L4;
   - Intrathecal injection of 1% ropivacaine 1.5 mL mixed with 10% glucose to 3 mL;
   - Epidural catheter insertion and fixation;
   - Adjust anesthesia level to T6.
6. **Surgery and Intervention:**
   - All cesarean deliveries will be performed via lower uterine segment vertical incision under combined spinal-epidural anesthesia.
   - **Intervention at umbilical cord clamping:**
     - **Experimental Group (E):** Intravenous S-Ketamine 0.5 mg/kg over 60±10 seconds. Postoperative PCIA with S-Ketamine 0.5 mg/kg + sufentanil 4 μg/kg in 200 mL saline (background 2 mL/h, bolus 3 mL, lockout 15 min, max 14 mL/h).
     - **Control Group (C):** Equivalent volume of saline. Postoperative PCIA with sufentanil 4 μg/kg in 400 mL saline (same settings).
   - Rescue analgesia: Tramadol 50 mg IV for NRS ≥4, repeatable after 4 hours.
7. **Postoperative Follow-up:**
   - Assess and record primary and secondary outcomes at specified time points.
8. **Outcome Measures:**
   - **Primary Outcome:** Highest pain NRS score in 24 hours postoperatively.
   - **Secondary Outcomes:**
     - Pressure pain thresholds at 0.5h and 24h;
     - Pressure pain tolerance at 0.5h and 24h;
     - Highest rest incision, visceral, and moving pain scores at 0-6h, 6-12h, 12-24h;
     - Serum CRP at 24h;
     - Time to first PCIA press;
     - PCIA press counts and drug consumption at 0-6h, 6-12h, 12-24h;
     - Tramadol consumption;
     - Anxiety/depression incidence on postoperative day 2;
     - Sleep disturbance incidence on postoperative day 2;
     - S-Ketamine-related adverse events (e.g., drowsiness, agitation, nausea/vomiting, hypertension, tachycardia).
9. **Scoring Systems:**
   - **Pain:** Numerical Rating Scale (NRS) 0-10 (0: no pain; 1-3: mild; 4-6: moderate; 7-10: severe).
   - **Pressure Pain Threshold/Tolerance:** Measured using a pressure algometer on the dominant forearm (three points, averaged).
   - **GAD-7, EPDS, PSQI:** Standard scoring methods.

**(7) Drug Storage, Dispensing, and Inventory:**

- Drugs will be stored and dispensed by designated personnel.
- PCIA pumps will be assigned sequentially and uniformly.

**(8) Concomitant Medications:**

- No additional analgesics allowed during the study.
- Necessary medications for comorbidities must be recorded.

**(9) Time Points:**

- T0: Preoperative;
- T1: 0.5h postoperative;
- T2: 24h postoperative.

**(10) Sample Size Calculation:**

- Based on preliminary data (NRS 6.5±0.9 vs. 4.0±1.0), α=0.05, power=0.9, 45/group + 10% dropout → 50/group (total 100).

**(11) Study Termination Criteria:**

- Early termination to protect participant safety or ensure data quality.

**(12) Adverse Events:**

- Defined as any untoward medical occurrence during the study.
- Managed per protocol (e.g., beta-blockers for tachycardia, antiemetics for nausea).
- Serious adverse events reported within 24 hours.

**(13) Statistical Analysis:**

- SPSS 22.0 for data analysis.
- Normal data: Mean±SD (t-test); non-normal: Median (IQR) (Mann-Whitney U); categorical: Chi-square. P<0.05 significant.

**(14) Unblinding and Emergency Unblinding:**

- Unblinding after database lock.
- Emergency unblinding permitted for severe adverse events.

**(15) Data Management:**

- CRFs completed accurately and archived.

**(16) Quality Control and Assurance:**

- Protocol training for all staff.
- Strict adherence to study procedures.
- Regular data verification.

**(17) Technical Route:**

- Participant screening → Baseline measurements → Anesthesia/surgery → Intervention → Postoperative assessments → Data analysis.

**(18) Key Technologies:**

1. S-Ketamine as a novel anesthetic for CD and its effects on pain hypersensitivity.
2. Serum CRP to explore anti-inflammatory mechanisms.

**(19) Feasibility Analysis:**

1. Theoretical: NMDA receptor role in pain hypersensitivity is well-established.
2. Team: Experienced in clinical research and data analysis.
3. Cases: Sufficient CD volume at our tertiary hospital.

**(20) Innovations:**

1. Novel use of S-Ketamine for CD pain hypersensitivity.
2. Mechanistic and clinical exploration of anti-inflammatory effects.

### IV. Annual Research Plan and Expected Outcomes

**(1) Annual Plan:**

- 2024.01-2024.02: Literature review, reagent procurement, team training, CRF preparation.
- 2024.2-2024.10: Participant recruitment, data collection (n=100).
- 2024.10-2024.12: Data analysis.
- 2025.01-2025.03: Manuscript writing, project closure.

**(2) Expected Outcomes:**

1. Confirm S-Ketamine’s efficacy in reducing postoperative pain after CD.
2. Publish at least one high-quality paper.
3. Conduct 1-2 continuing education programs.
4. Train at least one researcher.

### V. Research Foundation

**(1) Existing Work:**

1. Our department is a regional key discipline with extensive research experience.
2. PI has expertise in perioperative pain management (e.g., previous provincial project on ultrasound-guided CPSI).
3. Preliminary data (n=10) show S-Ketamine improves postoperative analgesia and reduces CRP.
